# Satisfaction and Quality of Life After Septorhinoplasty: Comparing Outcomes in Posttraumatic and Nontraumatic Cases Using the FACE-Q and NOSE Questionnaire

**DOI:** 10.1093/asjof/ojag177

**Published:** 2026-09-21

**Authors:** Nadia Sadok, Marius Mischke, Tobias Peter Bastian, Stefan Hansen, Stefan Mattheis, Stephan Lang, Kerstin Stähr

## Abstract

**Background:**

Functional septorhinoplasty (fSRP) aims to improve both nasal function and patient satisfaction.

**Objectives:**

This study compared postoperative satisfaction, nasal function, and quality of life after fSRP between patients with and without a history of nasal trauma using validated patient-reported outcome measures.

**Methods:**

A cross-sectional cohort of 71 patients (22 posttraumatic, 49 nontraumatic) who underwent fSRP between 2020 and 2024 at a tertiary referral center completed the Facial Assessment and Cosmetic Enhancement Quality of Life Questionnaire (FACE-Q), Nasal Obstruction Symptom Evaluation scale (NOSE), and World Health Organization Quality of Life—BREF questionnaire. Patients were grouped by history of nasal trauma, and multivariate linear regression was performed to assess factors influencing satisfaction and nasal function.

**Results:**

Patients with a history of nasal trauma reported significantly higher satisfaction on FACE-Q subscales “satisfaction with nose” (*β* = 12.12, *P* = .037) and “satisfaction with nostrils” (*β* = 17.01, *P* = .033) compared with nontrauma patients. Participants in a relationship scored higher on the subscales “satisfaction with nose” and “satisfaction with facial appearance” (*β*_adjusted_ = 13.47, *P* = .005 and *β*_adjusted_ = 11.80, *P* = .005, respectively). Smokers reported significantly higher nasal obstruction symptoms (*β* = 23.97, *P* = .002) on the NOSE scale. No significant difference in functional outcomes (NOSE score) was found between trauma and nontrauma groups.

**Conclusions:**

In this cohort, a history of nasal trauma and being in a relationship were associated with higher postoperative FACE-Q scores following fSRP. Individuals with previous nasal trauma reported greater satisfaction with their nose and nostrils, whereas those in a relationship demonstrated higher satisfaction with both their nose and overall facial appearance.

**Level of Evidence:**

3 (Therapeutic)
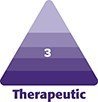

In the realm of aesthetic and reconstructive surgery, patient-reported outcome measures (PROMs) have emerged as indispensable tools for evaluating surgical outcomes from the patient's perspective.^1^ Among these, the Facial Assessment and Cosmetic Enhancement Quality of Life Questionnaire (FACE-Q) stands out for its comprehensive assessment of patients’ satisfaction and quality of life postsurgery, particularly in the context of facial procedures.^2,3^ This paper focuses on functional septorhinoplasty (fSRP), a procedure that has witnessed a significant increase in demand over the years, driven by the desire not only to correct functional impairments related to nasal breathing but also to enhance aesthetic appearance.^4^

The rising trend in cosmetic procedures can be attributed to several factors, with social media playing a pivotal role. Platforms such as Instagram and TikTok have not only changed the way individuals perceive beauty but have also heightened awareness and acceptance of cosmetic surgery.^5,6^ This shift in perception has contributed to a surge in the number of people seeking aesthetic enhancements, making the need for effective outcome measures like the FACE-Q more critical than ever.^5^ Because fSRP gains popularity for its dual benefits—improving nasal function while also addressing cosmetic concerns—this study seeks to delve into the satisfaction levels and quality of life of patients undergoing fSRP, particularly those with a history of nasal trauma. By employing the FACE-Q questionnaire, this research aims to provide valuable insights into the impact of posttraumatic fSRP, with the goal of informing future practices and enhancing patient care in the field of septorhinoplasty.^7^

Patient satisfaction following aesthetic and fSRP is influenced not only by surgical outcomes but also by psychosocial factors. Psychological instability has been associated with increased sensitivity to perceived physical imperfections and lower postoperative satisfaction, whereas psychological resilience and stable interpersonal relationships may contribute to improved self-perception and quality of life after surgery.^8-10^ These findings suggest that the impact of septorhinoplasty extends beyond anatomical correction and is closely linked to psychological and social well-being. Patients with nasal trauma frequently present with both functional impairment and aesthetic deformity, which may lead to lower preoperative satisfaction compared with nontrauma patients. As a result, this population may experience greater subjective benefit after surgery. However, comparative evidence regarding postoperative satisfaction and quality of life between trauma-related and nontrauma septorhinoplasty patients remains limited. Therefore, the aim of this study was to compare postoperative patient-reported satisfaction, nasal function, and quality of life between patients with and without a history of nasal trauma using validated PROMs. We hypothesized that patients undergoing fSRP after nasal trauma would report higher postoperative satisfaction than patients without previous nasal fractures.

## METHODS

### Study Design and Participants

This cross-sectional retrospective study included adult patients (≥18 years) previously submitted to fSRP between 2020 and 2024 at a tertiary referral center (University Hospital Essen, Department of Otorhinolaryngology—Head and Neck Surgery). Written consent for participation was obtained from each patient who chose to participate. The study was approved by the medical ethics committee (approval no. 23-11411-BO).

### Procedure

Using ICD codes, a search through the electronic medical system was performed. In total, 727 patients were identified who fit the search criteria. Patients who underwent other surgeries than fSRP, for example, septoplasty or nasal reposition for acute nasal fractures, were excluded from this study. First, all patients that underwent fSRP between January 2020 and April 2024 were contacted in writing between January 2024 and October 2024 and asked to participate. Patients who agreed to participate were asked to complete a medical history form, documenting baseline characteristics and details about any past nasal trauma. Besides these questions, the participants were asked to fill out 3 PROMs: the FACE-Q, the Nasal Obstruction Symptom Evaluation scale (NOSE), and the World Health Organization Quality of Life—BREF (WHOQOL-BREF) questionnaire. A reminder was sent after 4 weeks. This time the patients could decide between the paper form and a digital form, including a QR code that was printed on the reminder letters. Questionnaires were completed once at varying postoperative time points after surgery. No standardized preoperative PROM assessment was available for the retrospective cohort.

### Patient-Reported Outcome Measures

To assess patient satisfaction and quality of life after fSRP, we employed 2 validated PROMs: the FACE-Q Rhinoplasty Module and the NOSE. These PROMS are invaluable for assessing the results of aesthetic satisfaction, as well as functional nasal breathing following fSRP, from the patient's perspective.

#### Facial Assessment and Cosmetic Enhancement Quality of Life Questionnaire

The FACE-Q questionnaire comprises various subscales aimed at measuring perceived quality of life, aesthetic outcomes, psychosocial impact, and potential limitations before and after treatment. This provides physicians with the ability to assess treatment and surgical outcomes from the patient perspective following surgery. Raw FACE-Q responses were converted into Rasch-transformed scores according to the FACE-Q scoring guidelines and subsequently transformed to a 0 (worst) to 100 (best) scale for analysis.^2,7^

#### Nasal Obstruction Symptom Evaluation

This questionnaire assesses nasal function. The questionnaire consists of 5 questions, which can be answered with “not a problem” (1 point), “very mild problem” (2 points), “moderate problem” (3 points), and “severe problem” (4 points). The questions were “nasal congestion or stuffiness,” “nasal blockage or obstruction,” “trouble breathing through my nose,” “trouble sleeping,” and “unable to get enough air through my nose during exercise or exertion.” The points collected were then converted into a score from 0 to 100, with 0 corresponding to no complaints and 100 to the maximum complaints.^11,12^

#### World Health Organization Quality of Life—BREF

WHOQOL-BREF is a shortened version of the WHOQOL-100 quality of life assessment, developed by the World Health Organization (WHO). It is a psychometric instrument designed to evaluate an individual's perceived general quality of life across multiple domains: “physical health,” “psychological health,” “social relationships,” and “environment.” In addition, there are 2 general items: overall evaluation of quality of life and overall evaluation of health. The WHOQOL-BREF consists of 26 questions, and each item is rated on a scale from 0 to 5, which corresponds to a 5-point ordinal scale. These scores are subsequently converted linearly into a range from 0 to 100.^13^

### Definitions

Age was calculated in years. BMI was calculated in kilograms per square meters, and smoking status was rated either “yes” or “no.” fSRP was performed in either a closed or an open approach. In the latter, an inverted-V transcolumellar incision was made in all cases. All participants with a history of nasal trauma that was clinically and/or radiologically confirmed as a nasal structural fracture by a physician were categorized as posttraumatic cases. Relationship status was categorized as patients being in a steady relationship vs being single. Patients were also asked whether they had ever received psychological treatment (history of mental health treatment) and whether they currently had a mental illness (mental health issues now).

### Outcomes

The primary outcomes of this study were the following FACE-Q subscales: “satisfaction with the nose,” “satisfaction with the nostrils,” “appearance-related psychosocial distress,” “satisfaction with facial appearance overall,” “psychological well-being,” and “social confidence.” The NOSE questionnaire was considered the secondary outcome.

### Statistical Analyses

For participants’ characteristics, descriptive statistics were used. Continuous variables were presented by means and standard deviations (SDs) for normally distributed data and by medians and interquartile ranges (IQRs) for non-normally distributed data. Nominal variables were described by counts and proportions. FACE-Q and NOSE scores were presented as means (SD). Participant characteristics and FACE-Q scores were additionally stratified according to history of nasal trauma (traumatic vs nontraumatic) and surgical approach (open vs closed fSRP). Between-group comparisons were performed using the χ^2^ test for categorical variables and the Mann–Whitney *U* test for continuous variables. Linear regression analyses were established to analyze which factors were related to the FACE-Q scores. Based on the literature and institutional experience, we chose to examine the following patient characteristics: gender (female/male), age (in years), surgical approach (open vs closed), posttraumatic (yes/no), BMI, smoking (yes/no), relationship status (relationship/single), ever received psychological treatment (yes/no), current mental illness (yes/no). Besides that, we examined the following WHO scores: “physical health,” “psychological health,” “social relationships,” “environment,” and the general items “evaluation of quality of life” and “evaluation of health.” First, univariate linear analyses were carried out for each FACE-Q subscale and the NOSE questionnaire for the variables mentioned. If the significance was *P* ≤ .1, the variable was included in the multivariate linear analysis. For *P*-values >.1, the variable was excluded from the model. A 2-sided *P*-value of <.05 was considered statistically significant.

All statistical analyses were performed using IBM SPSS Statistics for Windows, Version 29.0 (IBM Corp., Armonk, NY).

### Post Hoc Sensitivity Power Analysis

Given the final sample size of 71 patients, with 22 patients in the posttraumatic group and 49 patients in the nontraumatic group, we performed a sensitivity power analysis. Assuming a 2-sided *α* of .05 and 80% power, the study was powered to detect a standardized mean difference of ∼0.73 between groups. Based on the observed SD of the FACE-Q “satisfaction with nose” subscale, this corresponds to a detectable difference of ∼17 points. Therefore, the study was sufficiently powered to detect moderate-to-large differences between groups but may have been underpowered to identify smaller differences.

## RESULTS

In total, we identified 727 patients that underwent fSRP in our clinic between January 2020 and April 2024 that fulfilled the criteria. All 727 were invited to participate in this study. Out of 727 patients, 106 (15%) could not be contacted because the letters could not be delivered because of incorrect or incomplete address and telephone information. Of the remaining 621 contactable patients, 71 responded, resulting in a response rate of 11%. Ten patients (14%) used the QR code and filled out the digital questionnaires. The time between surgery and the interview ranged from 3 months to 5 years, with a mean follow-up of 19.4 months. Seven patients (10%) had a minimum follow-up period of 3 months. Out of the 71 patients, 22 (30.99%) reported a past nasal trauma with fracture. The remaining 49 patients (69.01%) were defined as nontraumatic. Out of 22 traumatic patients, 9 fractures occurred during childhood (40.9%). Six out of 22 (27.3%) patients had radiological imaging to confirm the fracture; all other fractures were clinically diagnosed.

### Characteristics

The mean (SD) age for the total group was 33.8 (12). The mean (SD) BMI was 24.8 (3.8), and 56.3% of participants were female. The surgical technique used was roughly equally divided, with 35 procedures (49.3%) performed using an open approach and 36 procedures (50.7%) using a closed approach. General health-related quality of life was assessed using the WHOQOL-BREF questionnaire. The mean (SD) score for the “physical health domain” was 68.5 (19.3), whereas “psychological health” reached a mean of 65.2 (18.4). The domains “social relationships” and “environment” yielded comparable scores of 67.8 (24.3) and 70.7 (16.3), respectively. In addition, 2 individual items were assessed: “subjective quality of life,” with a mean score of 3.7 (0.9) and “satisfaction with health,” with a mean score of 3.4 (1.0). Patients with a history of nasal trauma were more frequently male (68.2% vs 30.8%, *P* = .005) and more often underwent a closed surgical approach (68.2% vs 38.5%, *P* = .024) than patients without a history of nasal trauma. Table 1 presents the participant characteristics stratified by history of nasal trauma, including WHO scores. Participants undergoing open and closed fSRP were comparable in terms of age, BMI, sex distribution, smoking status, relationship status, and history of mental health treatment (all *P* > .05). See Table 2 for participant characteristics stratified by surgical approach.

**Table 1.** Participant Characteristics Stratified by History of Nasal Trauma

	Total *n* = 71 (100%)	Traumatic total *n* = 22 (30.9%)	Nontraumatic *n* = 39 (54.9%)	*P*-value
Age (mean, SD)	33.8 (12)	34.5 (10.8)	33.9 (13.2)	.665
BMI (mean, SD)	24.8 (3.8)	24.0 (3.7)	25.5 (3.9)	.769
Gender				
Female	40 (56.3)	7 (31.8)	27 (69.2)	.005^a^
Male	31 (43.7)	15 (68.2)	12 (30.8)	
Surgical technique				
Open	35 (49.3)	7 (31.8)	24 (61.5)	.024^a^
Closed	36 (50.7)	15 (68.2)	15 (38.5)	
Smoking				
Yes	16 (22.5)	5 (22.7)	8 (20.5)	.839
No	53 (74.6)	17 (77.3)	31 (79.5)	
Missing	2 (2.8)	—	—	
Relationship status				
Single	35 (49.3)	7 (31.8)	21 (53.8)	.291
In a partnership	31 (43.7)	13 (59.1)	16 (41.0)	
Not specified	3 (4.2)	1 (4.5)	2 (5.1)	
Missing	2 (2.8)	—	—	
History of mental health treatment				
Yes	17 (23.9)	5 (22.7)	9 (23.1)	.728
No	47 (66.2)	16 (72.7)	26 (66.7)	
Not specified	6 (8.5)	1 (4.5)	4 (10.3)	
Missing	1 (1.4)	—	—	
Current mental health issues				
Yes	10 (14.1)	4 (18.2)	5 (12.8)	.765
No	57 (80.3)	17 (77.3)	33 (84.6)	
Not specified	3 (4.2)	1 (4.5)	1 (2.6)	
Missing	1 (1.4)	—	—	
Comorbidities				
Yes	15 (21.1)	5 (22.7)	8 (20.5)	.839
No	55 (77.5)	17 (77.3)	31 (79.5)	
Missing	1 (1.4)	—	—	
WHO domains, mean (SD)				
Physical health	68.5 (19.3)	69.3 (21.7)	67.8 (19.2)	.759
Psychological health	65.2 (18.4)	68.4 (22.0)	64.2 (15.9)	.164
Social relationships	67.8 (24.3)	69.7 (25.4)	67.3 (23.5)	.821
Environment	70.7 (16.3)	70.0 (17.2)	72.0 (16.1)	.352
Subjective quality of life	3.7 (0.9)	3.7 (0.9)	3.6 (1.0)	.848
Satisfaction with health	3.4 (1.0)	3.6 (1.1)	3.3 (1.1)	.805

Continuous variables are presented as mean (SD) and categorical variables as *n* (%). Comorbidities included cardiopulmonary disease, diabetes mellitus, and thyroid disease. WHOQOL-BREF domain scores were transformed to a 0 to 100 scale, with higher scores indicating better quality of life. Group comparisons were performed using Pearson's χ^2^ tests for categorical variables and independent *t*-tests for continuous variables. Statistical significance was defined as *P* < .05.

SD, standard deviation; WHOQOL-BREF, World Health Organization Quality of Life—Brief Version.

^a^Statistically significant difference (*P* < .05).

**Table 2.** Participant Characteristics Stratified by Open vs Closed Functional Septorhinoplasty

Characteristics	Total *n* = 71 (100%)	Open *n* = 35 (49.3%)	Closed *n* = 36 (50.7%)	*P*-value
Age (mean, SD)	33.8 (12)	35.9 (13.7)	31.8 (9.8)	.130
BMI (mean, SD)	24.8 (3.8)	24.9 (3.6)	24.8 (4.1)	.909
Gender				
Female	40 (56.3)	20 (57.1)	20 (55.6)	.542
Male	31 (43.7)	15 (42.9)	16 (44.4)	
Traumatic				
Yes	22 (30.9)	7 (20.0)	15 (41.7)	.024^a^
No	39 (54.9)	24 (68.6)	15 (41.7)	
Smoking				
Yes	16 (22.5)	8 (22.9)	8 (22.2)	.586
No	53 (74.6)	26 (74.3)	27 (75.0)	
Missing	2 (2.8)	—	—	
Relationship status				
Single	35 (49.3)	20 (57.1)	15 (41.7)	.286
In a partnership	31 (43.7)	12 (34.3)	19 (52.8)	
Not specified	3 (4.2)	1 (2.9)	2 (5.6)	
Missing	2 (2.8)	2 (5.7)	—	
History of mental health treatment				
Yes	17 (23.9)	6 (17.1)	11 (30.6)	.448
No	47 (66.2)	25 (71.4)	22 (61.1)	
Not specified	6 (8.5)	3 (8.6)	3 (8.3)	
Missing	1 (1.4)	1 (2.9)	—	
Current mental health issues				
Yes	10 (14.1)	3 (8.6)	7 (19.4)	.361
No	57 (80.3)	30 (85.7)	27 (75.0)	
Not specified	3 (4.2)	1 (2.9)	2 (5.6)	
Missing	1 (1.4)	1 (2.9)	—	
Comorbidities				
Yes	15 (21.1)	7 (20.0)	8 (22.2)	.550
No	55 (77.5)	27 (77.1)	28 (77.8)	
Missing	1 (1.4)	—	—	
WHO domains, mean (SD)				
Physical health	68.5 (19.3)	67.8 (19.8)	69.2 (19.0)	.923
Psychological health	65.2 (18.4)	65.2 (17.1)	65.2 (19-8)	.922
Social relationships	67.8 (24.3)	64.7 (27.1)	70.7 (21.3)	.576
Environment	70.7 (16.3)	70.1 (16.8)	71.2 (16.0)	.681
Subjective quality of life	3.7 (0.9)	3.6 (1.0)	3.8 (0.9)	.805
Satisfaction with health	3.4 (1.0)	3.3 (1.2)	3.4 (0.9)	.119

Continuous variables are presented as mean (SD) and categorical variables as *n* (%). Comorbidities included cardiopulmonary disease, diabetes mellitus, and thyroid disease. WHOQOL-BREF domain scores were transformed to a 0 to 100 scale, with higher scores indicating better quality of life. Group comparisons were performed using Pearson's χ^2^ tests for categorical variables and independent *t*-tests for continuous variables.

SD, standard deviation; SRP, septorhinoplasty; WHOQOL-BREF, World Health Organization Quality of Life—Brief Version.

^a^Statistically significant difference (*P* < .05).

### Primary and Secondary Outcomes

On average, patients reported a mean (SD) score of 52.8 (23.0) for “satisfaction with the nose” and 61.8 (30.6) for “satisfaction with the nostrils.” “Satisfaction with facial appearance overall” reached a mean score of 52.1 (20.3). The subscale “appearance-related psychosocial distress” yielded a mean score of 28.4 (24.5), indicating comparatively lower distress levels. Regarding psychosocial domains, the subscale “psychological well-being” showed a mean score of 68.0 (24.5), whereas “social confidence” was rated at 60.9 (24.2). An overview of all FACE-Q subscale scores for the total cohort, stratified by history of nasal trauma (traumatic vs nontraumatic) and by surgical approach (open vs closed fSRP), is presented in Tables 3 and 4. The median (IQR) NOSE score was 40 (47.5).

**Table 3.** Comparison of FACE-Q Scores Between Patients Undergoing Functional Septorhinoplasty for Traumatic and Nontraumatic Indications

	Total group *n* = 71 (100%)		Traumatic *n* = 22 (30.9%)		Nontraumatic *n* = 39 (54.9%)		Statistics
Subscale	*n*	Mean (SD)	*n*	Mean (SD)	*n*	Mean (SD)	*P*-value
FACE-Q nose	69	52.8 (23.0)	22	63.8 (18.8)	38	45.6 (23.2)	.003^a^
FACE-Q nostrils	71	61.8 (30.6)	22	75.6 (22.7)	39	51.5 (30.8)	.003^a^
FACE-Q appearance-related psychosocial distress	70	28.4 (24.5)	22	23.1 (25.1)	38	30.8 (25.6)	.226
FACE-Q satisfaction with facial appearance overall	69	52.1 (20.3)	22	61.6 (19.7)	38	47.0 (20.5)	.020^a^
FACE-Q psychological well-being	70	68.0 (24.5)	22	76.6 (21.6)	38	65.7 (25.7)	.109
FACE-Q social confidence	71	60.9 (24.2)	22	63.3 (24.2)	39	59.3 (25.1)	.593

Values are presented as mean (standard deviation, SD) of Rasch-transformed FACE-Q scores converted to a 0 to 100 scale. Higher scores indicate better outcomes for all FACE-Q scales except the Appearance-Related Psychosocial Distress scale, for which lower scores indicate less distress. *n* denotes the number of completed questionnaires for each subscale. Group comparisons between patients with traumatic and nontraumatic nasal deformities were performed using the Mann–Whitney *U* test.

FACE-Q, Facial Assessment and Cosmetic Enhancement—Quality of Life.

^a^Statistically significant difference (*P* < .05).

**Table 4.** Comparison of FACE-Q Scores Between Patients Undergoing Functional Septorhinoplasty for Open vs Closed Functional Septorhinoplasty

	Total group *n* = 71 (100%)		Open approach *n* = 35 (49.3%)		Closed approach *n* = 36 (50.7%)		Statistics
Subscale	*n*	Mean (SD)	*n*	Mean (SD)	*n*	Mean (SD)	*P*-value
FACE-Q nose	69	52.8 (23.0)	33	50.4 (23.5)	36	55.0 (22.7)	.407
FACE-Q nostrils	71	61.8 (30.6)	35	58.6 (30.1)	36	65.0 (31.1)	.353
FACE-Q appearance-related psychosocial distress	70	28.4 (24.5)	34	29.3 (24.0)	36	27.5 (25.3)	.615
FACE-Q satisfaction with facial appearance overall	69	52.1 (20.3)	35	51.0 (20.6)	34	53.2 (20.3)	.524
FACE-Q psychological well-being	70	68.0 (24.5)	34	65.6 (25.9)	36	71.5 (22.5)	.416
FACE-Q social confidence	71	60.9 (24.2)	35	55.5 (26.4)	36	66.1 (20.8)	.099

Values are presented as mean (standard deviation, SD) of Rasch-transformed FACE-Q scores converted to a 0 to 100 scale. Higher scores indicate better outcomes for all FACE-Q scales except the Appearance-Related Psychosocial Distress scale, for which lower scores indicate less distress. *n* denotes the number of completed questionnaires for each subscale. Group comparisons between patients who underwent an open vs a closed approach were performed using the Mann–Whitney *U* test.

FACE-Q, Facial Assessment and Cosmetic Enhancement—Quality of Life.

### Multivariate Linear Regression Analyses

Participants with a history of nasal trauma had significantly higher FACE-Q scores for the subscales “satisfaction with nose” and “satisfaction with nostrils” (*β*_adjusted_ = 12.12, *P* ≤ .037 and *β*_adjusted_ = 17.01, *P* ≤ .033, respectively). Those with a history of nasal trauma also scored higher on the subscale “face appearance overall,” although this was not statistically significant in the multivariate model (*β*_adjusted_ = 9.04, *P* ≤ .070).

There was a statistically significant correlation between relationship status and “satisfaction with facial appearance.” Participants who were in a relationship scored higher on the subscales “satisfaction with nose” and “satisfaction with facial appearance” (*β*_adjusted_ = 13.47, *P* ≤ .005 and *β*_adjusted_ = 11.80, *P* ≤ .005, respectively).

Higher BMI correlated with lower satisfaction on the FACE-Q “satisfaction with nostrils” subscale but was not statistically significant (*β*_adjusted_ = −13.79, *P* ≤ .082).

The WHOQOL domain “psychological health” was statistically significantly associated with the FACE-Q subscale “psychological well-being” (*β*_adjusted_ = .56, *P* ≤ .045). Other domains of the WHOQOL were not associated with any of the FACE-Q subscales.

As for the NOSE questionnaire, the analyses showed that smokers had more symptoms of nasal obstruction compared with nonsmokers (*β*_adjusted_ = 23.97, *P* ≤ .002). There was no statistically significant difference in NOSE outcomes between patients with past nasal trauma and patients without. Neither surgical technique nor demographic factors such as age, gender, or previous psychological treatments were statistically significantly associated with any of the FACE-Q subscales or NOSE questionnaire. Table 5 presents the results of the multivariate linear regression analyses. For the univariate linear regression analyses, please see Supplemental Table 1.

**Table 5.** Multivariate Regression Analyses of Predictors Associated With Functional and Patient-Reported Outcome Measures Following Functional Septorhinoplasty

Questionnaire	Variable	*β* (95% CI)	*P*-value
NOSE	Smoking (yes/no)	23.97 (9.50-38.45)	.002^a^
	Satisfaction with health	−6.13 (−13.02 to 0.75)	.080
FACE-Q satisfaction with nose	Relationship status	13.47 (4.21-22.72)	.005^a^
	Trauma (yes/no)	12.12 (0.78-23.46)	.037^a^
FACE-Q satisfaction with nostrils	BMI (<25/≥25)	−13.79 (−29.38 to 1.80)	.082
	Relationship status	10.92 (−1.74 to 23.57)	.089
	Trauma (yes/no)	17.01 (1.44-32.57)	.033^a^
FACE-Q appearance-related psychosocial distress	Relationship status	−10.06 (−18.47 to −1.64)	.020^a^
	WHO physical health domain	−.57 (−1.06 to −0.07)	.026^a^
	WHO psychological domain	−.67 (−1.13 to −0.22)	.005^a^
	WHO environment domain	.58 (0.03-1.12)	.038^a^
FACE-Q satisfaction with facial appearance overall	Relationship status	11.80 (3.71-19.88)	.005^a^
	Trauma (yes/no)	9.04 (−0.78 to 18.87)	.070
FACE-Q psychological well-being	WHO psychological domain	.56 (0.01-1.11)	.045^a^
FACE-Q social confidence	WHO social relationships domain	.32 (−0.06 to 0.69)	.095
	Subjective quality of life	7.49 (−0.33 to 15.31)	.060

Regression coefficients (*β*) are presented with their corresponding 95% CIs. Only variables with *P* < .10 in the univariate analyses were included in the multivariate models. FACE-Q scores are presented as Rasch-transformed scores converted to a 0 to 100 scale. Higher FACE-Q scores indicate better outcomes, except for the Appearance-Related Psychosocial Distress scale, for which lower scores indicate less distress.

NOSE, Nasal Obstruction Symptom Evaluation scale; WHO, World Health Organization.

^a^Statistically significant association (*P* < .05).

## DISCUSSION

The aim of this study was to identify factors influencing patient satisfaction and quality of life after fSRP, with a focus on previous nasal trauma. Our results, assessed using the FACE-Q, NOSE, and WHOQOL-BREF questionnaires, highlight several key findings. In this study, patients with a history of nasal trauma showed higher satisfaction with their nose and nostrils compared with nontrauma patients post-fSRP, which fits the study's hypothesis (*β*_adjusted_ = 12.12, *P* ≤ .037; *β*_adjusted_ = 17.01, *P* ≤ .033). Higher satisfaction among the participants with a history of trauma could possibly be explained by the fact that trauma patients seek to regain their original nasal appearance, which was altered by injury, whereas patients without previous trauma do not have an earlier aesthetic standard for comparison. The experience of losing an established appearance may heighten sensitivity to aesthetic changes, making even subtle postoperative improvements feel particularly rewarding.^14,15^ This goes hand in hand with findings from other studies, which also show higher levels of satisfaction among trauma patients who underwent rhinoplasty.^16^

As already shown in other studies, social relationships have an influence on self-image and psychological self-esteem. This could explain why patients in a relationship give higher values regarding their satisfaction with optical variables (“satisfaction with nose” *β*_adjusted_ = 13.47, *P* ≤ .005; “satisfaction with facial appearance overall” *β*_adjusted_ = 11.80, *P* ≤ .005) than patients who are single.^17-19^ The fact that the psyche has an influence on self-esteem and visual self-perception can also be seen in the WHO psychological domain scores. Patients who achieved higher values there also showed better values in the FACE-Q score regarding their psychological perception (*β*_adjusted_ = .56, *P* ≤ .045). In contrast, the other WHO domains showed no statistically significant influence. This suggests a stronger link between psychological quality of life and subjective well-being than with appearance parameters. Higher BMI correlated with lower satisfaction on the FACE-Q scales but was not statistically significant (FACE-Q nostrils *β*_adjusted_ = −13.79, *P* ≤ .082). The literature confirms that a high BMI is generally associated with lower satisfaction with external appearance. This suggests that the effect of high BMI does not influence satisfaction with the nose, possibly because of the fact that the external appearance of the nose is generally not affected by weight gain or higher body fat.^20,21^

Smoking showed a significant association with worse nasal obstruction symptoms (NOSE *β*_adjusted_ = 23.97, *P* ≤ .002), which can be explained because of its impact on mucous membranes and wound healing.^22,23^

In interpreting these findings, it is important to acknowledge that our study did not include a longitudinal preoperative and postoperative comparison, which limits the ability to measure within-patient change over time. Nevertheless, the obtained FACE-Q and NOSE values can be contextualized with data from the literature. For instance, Kandathil et al reported mean postoperative NOSE scores that are comparable to our cohort, supporting the external validity of our results.^24^

### Strengths and Limitations

This study is the first to use the German version of the validated FACE-Q Rhinoplasty module, enabling the evaluation of data from a German population with a culturally and linguistically adapted instrument.^7^ Furthermore, the application of internationally recognized and validated PROMs strengthens the clinical relevance and validity of the findings.

With a sample size of 71 patients, the study provides valuable insights into patient-reported outcomes after fSRP. However, the relatively small cohort limits the generalizability of the results and reduces the statistical power to detect smaller effects. Furthermore, the relatively small sample size in relation to the number of variables included in the regression analyses may have increased the risk of model overfitting. Consequently, some observed associations may be unstable and should be interpreted with caution. The retrospective study design is another limitation, because it restricts the ability to control data collection methods and increases the potential for recall bias and incomplete data. Prospective studies would allow for a more standardized assessment and the possibility to directly measure changes before and after surgery. Additionally, the single-center nature of the study may limit the generalizability of the results, because treatment techniques, patient populations, and standards of care can vary across different institutions.

Another important limitation is the relatively low response rate of ∼11%. This raises the possibility of selection bias, because patients who were either particularly satisfied or dissatisfied may have been more motivated to respond. Such bias could have influenced the distribution of satisfaction scores in our cohort. Considerable effort was made to improve participation; however, our institution serves a highly diverse patient population in the Ruhr metropolitan area, including many patients with a migration background and limited German language proficiency, who frequently attend consultations accompanied by translators. According to demographic data from the City of Essen, approximately one-third of the population has a migration background, and residents from more than 140 countries live in Essen.^25^ Although we do not possess exact data regarding German-speaking vs non-German-speaking patients within our cohort, language barriers may have negatively affected participation in this questionnaire-based follow-up study. Future prospective studies with higher participation rates and systematic preoperative and postoperative assessments are warranted to minimize this effect and to more accurately determine the magnitude of clinically meaningful improvement.

### Clinical Implications

Our data suggest that trauma patients benefit significantly more from surgery in terms of self-perceived satisfaction postoperative compared with nontrauma patients, whereas functional parameters and the overall impact on quality of life are unchanged in the groups. Clinically, this knowledge could be used to evaluate indications for surgery. Consideration should be given to more generous indications for trauma patients, because they clearly benefit from surgery in terms of patient satisfaction.

## CONCLUSIONS

Postoperative satisfaction following fSRP, as assessed by the FACE-Q, was influenced by both previous nasal trauma and relationship status in this cohort. Individuals with a history of nasal trauma reported significantly higher satisfaction with their nose and nostrils than those without previous trauma. Additionally, participants who were in a relationship reported greater satisfaction with their nasal appearance and overall facial appearance compared with single participants. These findings suggest that both clinical history and psychosocial factors may contribute to patient-reported satisfaction after fSRP.

## Supplemental Material

This article contains supplemental material located online at https://doi.org/10.1093/asjof/ojag177.

## Supplementary Material

ojag177_Supplementary_Data
