# Supplementary material for: Satisfaction and Quality of Life After Septorhinoplasty: Comparing Outcomes in Posttraumatic and Nontraumatic Cases Using the FACE-Q and NOSE Questionnaire
Source: Aesthet Surg J Open Forum. 2026 Sep 21;8:ojag177. doi: 10.1093/asjof/ojag177 (PMC13650683; doi:10.1093/asjof/ojag177)
Supplement: Supplementary file 1 [file ojag177_supplementary_data.docx]

**Supplementary Table 1: Univariate linear regression analyses of predictors associated with functional and patient-reported outcomes following functional septorhinoplasty.**

| Questionnaire | Variable | ß (95% CI) | P-Value |
| --- | --- | --- | --- |
| NOSE | Age | 0.26 (0.96; 0.34) | 0.343 |
|  | Gender | 3.22 (-9.90; 16.34) | 0.626 |
|  | Surgical technique (open/closed) | 2.24 (-10.80; 15.27) | 0.733 |
|  | BMI | 1.04 (-0.66; 2.74) | 0.227 |
|  | BMI (<25/>=25) | 6.20 (-6.85; 19.24) | 0.347 |
|  | BMI (<30/>=30) | 9.15 (-12.61; 30.91) | 0.404 |
|  | Relationship status | -2.16 (-13.47; 9.51) | 0.704 |
|  | History of mental health treatment | 9.10 (-0.74; 18.94) | 0.069* |
|  | Mental health issues now | 2.03 (-10.62; 14.68) | 0.750 |
|  | Smoking (yes/no) | 25.25 (10.96; 39.55) | < 0.001* |
|  | Trauma (yes/no) | 2.52 (-11.79; 16.84) | 0.725 |
|  | WHO Physical health Domain | -0.43 (-0.75; -0.10) | 0.011* |
|  | WHO Psychological Domain | -0.08 (-0.44; 0.28) | 0.672 |
|  | WHO Social relationships Domain | -0.07 (-0.35; 0.21) | 0.622 |
|  | WHO Environment Domain | -0.20 (-0.60; 0.21) | 0.342 |
|  | Subjective Quality of life | -3.26 (-10.39; 3.86) | 0.364 |
|  | Satisfaction with health | -9.97 (-15.76; -4.17) | 0.001* |
| Face-Q Satisfaction with Nose | Age | -0.07 (-0.53; 0.39) | 0.765 |
|  | Gender | 0.77 (-10.47; 12.01) | 0.891 |
|  | Surgical technique (open/closed) | 4.55 (-6.55; 15.65) | 0.416 |
|  | BMI | -1.93 (-3.33; -0.53) | 0.008* |
|  | BMI (<25/>=25) | -12.68 (-23.56; -1.80) | 0.023* |
|  | BMI (<30/>=30) | -10.09 (-28.73; 8.55) | 0.284 |
|  | Relationship status | 14.31 (5.54; 23.07) | 0.002* |
|  | History of mental health treatment | -3.30 (-11.89; 5.28) | 0.445 |
|  | Mental health issues now | -0.86 (-11.68; 9.96) | 0.874 |
|  | Smoking (yes/no) | 1.58 (-11.62; 14.78) | 0.812 |
|  | Trauma (yes/no) | 18.19 (6.56; 29.81) | 0.003* |
|  | WHO Physical health Domain | 0.44 (0.174; 0.709) | 0.002* |
|  | WHO Psychological Domain | 0.33 (0.04; 0.63) | 0.026* |
|  | WHO Social relationships Domain | 0.22 (-0.01; 0.44) | 0.062* |
|  | WHO Environment Domain | 0.23 (-0.11; 0.57) | 0.187 |
|  | Subjective Quality of life | 8.24 (2.47; 14.01) | 0.006* |
|  | Satisfaction with health | 7.87 (2.75; 12.99) | 0.003* |
| Face-Q Satisfaction with Nostrils | Age | -0.17 (-0.78; 0.44) | 0.580 |
|  | Gender | -3.19 (-17.87; 11.48) | 0.666 |
|  | Surgical technique (open/closed) | 6.37 (-8.13; 20.87) | 0.384 |
|  | BMI | -2.10 (-3.78; 0.22) | 0.029* |
|  | BMI (<25/>=25) | -18.79 (-32.89; -4.69) | 0.010* |
|  | BMI (<30/>=30) | -5.43 (-29.90; 19.04) | 0.659 |
|  | Relationship status | 11.71 (-0.62; 24.04) | 0.062* |
|  | History of mental health treatment | -2.94 (-14.28; 8.40) | 0.606 |
|  | Mental health issues now | 4.06 (-10.19; 18.31) | 0.572 |
|  | Smoking (yes/no) | -8.84 (-26.08; 8.41) | 0.310 |
|  | Trauma (yes/no) | 24.06 (9.03; 39.09) | 0.002* |
|  | WHO Physical health Domain | 0.66 (0.31; 1.00) | <0.001* |
|  | WHO Psychological Domain | 0.52 (0.14; 0.91) | 0.008* |
|  | WHO Social relationships Domain | 0.36 (0.05; 0.62) | 0.023* |
|  | WHO Environment Domain | 0.57 (0.14; 1.00) | 0.011* |
|  | Subjective Quality of life | 10.33 (2.74; 17.92) | 0.008* |
|  | Satisfaction with health | 9.95 (3.26; 16.64) | 0.004* |
| Face-Q Appearance-Related Psychosocial Distress | Age | 0.39 (-0.10; 0.87) | 0.114 |
|  | Gender | 7.83 (-3.93; 19.59) | 0.188 |
|  | Surgical technique (open/closed) | -1.82 (-13.61; 9.96) | 0.759 |
|  | BMI | 1.58 (0.08; 3.09) | 0.040* |
|  | BMI (<25/>=25) | 11.93 (0.37; 23.49) | 0.043* |
|  | BMI (<30/>=30) | 2.94 (-16.79; 22.66) | 0.767 |
|  | Relationship status | -9.58 (-19.57; 0.41) | 0.060* |
|  | History of mental health treatment | 9.88 (1.04; 18.73) | 0.029* |
|  | Mental health issues now | 8.30 (-3.03; 19.62) | 0.148 |
|  | Smoking (yes/no) | -1.96 (-16.30; 12.39) | 0.786 |
|  | Trauma (yes/no) | -7.68 (-21.311; 5.95) | 0.264 |
|  | WHO Physical health Domain | -0.61 (-0.88; -0.34) | <0.001* |
|  | WHO Psychological Domain | -0.77 (-1.03; -0.51) | <0.001* |
|  | WHO Social relationships Domain | -0.49 (-0.71; -0.28) | <0.001* |
|  | WHO Environment Domain | -0.47 (-0.82; -0.13) | 0.008* |
|  | Subjective Quality of life | -7.03 (-13.21; -0.84) | 0.027* |
|  | Satisfaction with health | -6.38 (-11.98; -0.78) | 0.026* |
| Face-Q satisfaction with facial appearance overall | Age | -0.05 (-0.46; 0.36) | 0.805 |
|  | Gender | 2.77 (-7.10; 12.65) | 0.577 |
|  | Surgical technique (open/closed) | 2.21 (-7.63; 12.04) | 0.656 |
|  | BMI | -0.99 (-2.27; 0.29) | 0.128 |
|  | BMI (<25/>=25) | -7.13 (-16.94; 2.68) | 0.151 |
|  | BMI (<30/>=30) | -6.18 (-22.47; 10.12) | 0.451 |
|  | Relationship status | 12.62 (4.88; 20.36) | 0.002* |
|  | History of mental health treatment | -6.09 (-14.00; 1.82) | 0.129 |
|  | Mental health issues now | -4.33 (-15.07; 6.41) | 0.424 |
|  | Smoking (yes/no) | 3.53 (-8.58; 15.65) | 0.562 |
|  | Trauma (yes/no) | 14.57 (3.73; 25.40) | 0.009* |
|  | WHO Physical health Domain | 0.48 (0.25; 0.71) | <0.001* |
|  | WHO Psychological Domain | 0.41 (0.16; 0.66) | 0.002* |
|  | WHO Social relationships Domain | 0.33 (0.14; 0.52) | 0.001* |
|  | WHO Environment Domain | 0.29 (-0.01; 0.58) | 0.059* |
|  | Subjective Quality of life | 6.66 (1.59; 11.73) | 0.011* |
|  | Satisfaction with health | 7.80 (3.46; 12.14) | <0.001* |
| Face-Q psychological Well-Being | Age | -0.21 (-0.69; 0.27) | 0.382 |
|  | Gender | -4.72 (-16.41; 6.97) | 0.423 |
|  | Surgical technique (open/closed) | 5.86 (-5.69; 17.40) | 0.315 |
|  | BMI | -1.19 (-2.72; 0.35) | 0.129 |
|  | BMI (<25/>=25) | -14.28 (-25.73; -2.83) | 0.015* |
|  | BMI (<30/>=30) | 3.44 (-16.25; 23.12) | 0.728 |
|  | Relationship status | 9.34 (-0.60; 19.27) | 0.065* |
|  | History of mental health treatment | -9.77 (-18.58; -0.96) | 0.030* |
|  | Mental health issues now | -11.17 (-22.29; -0.04) | 0.049* |
|  | Smoking (yes/no) | -2.33 (-16.65; 12.00) | 0.747 |
|  | Trauma (yes/no) | 10.89 (-2.15; 23.92) | 0.100 |
|  | WHO Physical health Domain | 0.54 (0.27; 0.82) | <0.001* |
|  | WHO Psychological Domain | 0.72 (0.45; 0.99) | <0.001* |
|  | WHO Social relationships Domain | 0.44 (0.22; 0.66) | <0.001* |
|  | WHO Environment Domain | 0.39 (0.04; 0.74) | 0.028* |
|  | Subjective Quality of life | 11.68 (6.02; 17.34) | <0.001* |
|  | Satisfaction with health | 9.38 (4.13; 14.64) | <0.001* |
| Face-Q Social Confidence | Age | -0.13 (-0.62; 0.35) | 0.589 |
|  | Gender | -6.70 (-18.12; 4.81) | 0.249 |
|  | Surgical technique (open/closed) | 10.63 (-0.62; 21.87) | 0.064* |
|  | BMI | -0.926 (-2.46; 0.61) | 0.233 |
|  | BMI (<25/>=25) | -9.12 (-20.74; 2.52) | 0.123 |
|  | BMI (<30/>=30) | 0.95 (-18.83; 20.73) | 0.924 |
|  | Relationship status | 9.30 (-0.19; 18.78) | 0.055* |
|  | History of mental health treatment | -2.69 (-11.75; 6.37) | 0.555 |
|  | Mental health issues now | -3.44 (14.82; 7.95) | 0.549 |
|  | Smoking (yes/no) | -4.63 (-18.37; 9.10) | 0.503 |
|  | Trauma (yes/no) | 4.04 (-9.20; 17.27) | 0.544 |
|  | WHO Physical health Domain | 0.34 (0.05; 0.63) | 0.024* |
|  | WHO Psychological Domain | 0.37 (0.06; 0.67) | 0.020* |
|  | WHO Social relationships Domain | 0.38 (0.16; 0.61) | 0.001* |
|  | WHO Environment Domain | 0.43 (0.08; 0.77) | 0.016* |
|  | Subjective Quality of life | 10.23 (4.41; 16.04) | <0.001* |
|  | Satisfaction with health | 5.63 (0.17; 11.08) | 0.043* |
| Regression coefficients (β) are presented with their corresponding 95% confidence intervals (95% CI). FACE-Q scores are presented as Rasch-transformed scores converted to a 0–100 scale. Higher FACE-Q scores indicate better outcomes, except for the Appearance-Related Psychosocial Distress scale, for which lower scores indicate less distress. Variables with p < 0.10 in the univariate analyses were considered for inclusion in the multivariate regression models. BMI = Body Mass Index; WHO = World Health Organization; NOSE = Nasal Obstruction Symptom Evaluation scale. *Association with p < 0.10. | | | |
